# Hepatic glycogen storage diseases are associated to microbial dysbiosis

**DOI:** 10.1371/journal.pone.0214582

**Published:** 2019-04-02

**Authors:** Karina Colonetti, Bruna Bento dos Santos, Tatiéle Nalin, Carolina Fischinger Moura de Souza, Eric W. Triplett, Priscila Thiago Dobbler, Ida Vanessa Doederlein Schwartz, Luiz Fernando Wurdig Roesch

**Affiliations:** 1 Post-Graduation Program in Genetics and Molecular Biology, Universidade Federal do Rio Grande do Sul, Porto Alegre, Rio Grande do Sul, Brazil; 2 Laboratory of Basic Research and Advanced Investigations in Neurosciences (BRAIN), Hospital de Clínicas de Porto Alegre, PortoAlegre, Rio Grande do Sul, Brazil; 3 Medical Genetics Service, Hospital de Clínicas de Porto Alegre, Porto Alegre, Rio Grande do Sul, Brazil; 4 Postgraduate Program in Medicine: Medical Sciences, Universidade Federal do Rio Grande do Sul, Porto Alegre, Rio Grande do Sul, Brazil; 5 Department of Microbiology and Cell Science, Institute of Food and Agricultural Sciences, University of Florida, Gainesville, FL, United States of America; 6 Interdisciplinary Research Center on Biotechnology-CIP-Biotec, Universidade Federal do Pampa, São Gabriel, Rio Grande do Sul, Brazil; "INSERM", FRANCE

## Abstract

**Introduction:**

The gut microbiome has been related to several features present in Glycogen Storage Diseases (GSD) patients including obesity, inflammatory bowel disease (IBD) and liver disease.

**Objectives:**

The primary objective of this study was to investigate associations between GSD and the gut microbiota.

**Methods:**

Twenty-four GSD patients on treatment with uncooked cornstarch (UCCS), and 16 healthy controls had their faecal microbiota evaluated through 16S rRNA gene sequencing. Patients and controls were ≥3 years of age and not on antibiotics. Faecal pH, calprotectin, mean daily nutrient intake and current medications were recorded and correlated with gut microbiome.

**Results:**

Patients’ group presented higher intake of UCCS, higher prevalence of IBD (n = 04/24) and obesity/overweight (n = 18/24) compared to controls (n = 0 and 06/16, respectively). Both groups differed regarding diet (in patients, the calories’ source was mainly the UCSS, and the intake of fat, calcium, sodium, and vitamins was lower than in controls), use of angiotensin-converting enzyme inhibitors (patients = 11, controls = 0; p-value = 0.001) multivitamins (patients = 22, controls = 01; p-value = 0.001), and mean faecal pH (patients = 6.23; controls = 7.41; p = 0.001). The GSD microbiome was characterized by low diversity and distinct microbial structure. The operational taxonomic unit (OTU) abundance was significantly influenced by faecal pH (r = 0.77; p = 6.8e-09), total carbohydrate (r = -0.6; p = 4.8e-05) and sugar (r = 0.057; p = 0.00013) intakes.

**Conclusions:**

GSD patients presented intestinal dysbiosis, showing low faecal microbial diversity in comparison with healthy controls. Those findings might be due to the disease *per se*, and/or to the different diets, use of UCSS and of medicines, and obesity rate found in patients. Although the main driver of these differences is unknown, this study might help to understand how the nutritional management affects GSD patients.

## Introduction

Hepatic Glycogen Storage Diseases (GSD) are genetic disorders caused by deficient activity of one of the enzymes involved in the glycogenolysis pathway. The global incidence is estimated at 1 case per 20,000–43,000 live births. The most common types of GSD are GSD I, GSD III and GSD Ixα [1].

In GSD I, glucose-6-phosphate cannot be dephosphorylated to free glucose. There are two major subtypes of GSDI: Ia (~80%), caused by mutations in the *G6PC* gene, and GSD Ib (~20%), caused by mutations in the *SLC37A4* gene. The proteins produced from *G6PC* (catalytic activity) and *SLC37A4* (transporter) work together [2]. GSD Ia involves glycogenolysis and gluconeogenesis, and the clinical manifestations are increased weight, hepatomegaly, failure to thrive, fasting hypoglycaemia, high lactate, hyperuricemia, nephromegaly and hyperlipidaemia [3]. In addition to the features presented in GSD Ia, GSD Ib also presents with susceptibility to recurrent infections, impaired neutrophil and monocyte function, and inflammatory bowel disease (Crohn’s-like IBD) [1].

Mutations in the *AGL* gene cause GSD type III, in which the defective glycogen debranching enzyme blocks glycogenolysis, stopping the conversion of glycogen to glucose-1-phosphate [4]. At the same time, gluconeogenesis is enhanced to help maintain endogenous glucose production. Hepatomegaly in type III GSD generally improves with age, but affected individuals may develop chronic liver disease (cirrhosis) and liver failure later in life [5].

GSD IX is caused by the inability of phosphorylase b kinase (PHKA) to break down the glycogen in liver and/or muscle cells. Type IXα glycogenosis is an X-linked disease caused by mutations in the alpha subunit of *PHKA*. The signs and symptoms typically begin in early childhood, but GSD IX is usually milder than the other types [6].

The treatment for the aforementioned types of GSD involves nutritional adjustments primarily, with the periodic and frequent administration of large amounts of uncooked cornstarch (UCCS) and restriction of simple carbohydrates [7] to maintain normoglycaemia and avoid glycogen storage. Usually, higher and frequent doses of UCCS are prescribed for type Ia patients and lower doses for type IX patients. The dose is adjusted according to weight and metabolic demand [8]. GSD III and IX patients may require a hyperproteic diet with fewer restrictions for simple sugars. Sometimes additional medications may be necessary.

During the last decades, our understanding of the human being has changed. We know now that the eukaryote cells encoded by our genome are not the only component of our body. Symbiont prokaryotic cells inhabiting many cavities of our body provide metabolic functions far beyond the scope of our own physiological capabilities [9]. These cells play an important role in health and disease states [10]. The gut microbes are the most studied human associated microbial communities and consists of trillions of microbes and millions of functional genes [11]. Healthy humans present a remarkable microbial diversity but with similar functions indicating that different microbial communities are associated with a healthy microbiome [12]. The gut microbiome can be influenced by diet, lifestyle, drugs and genetics of the host [13], and has been related to several features present in GSD patients including obesity, IBD and liver disease [14]. This work aimed to investigate possible associations between GSD and the gut microbiota.

## Methods

This study was a cross-sectional, observational convenience sampling study, which included 24 GSD patients (Ia = 15, Ib = 5, III = 1, IXα = 3) and 16 healthy controls. All patients were recruited from the outpatient clinics of the Medical Genetics Service at Hospital de Clínicas de Porto Alegre (MGS-HCPA), Brazil from Jan/2016 to May/2017. As inclusion criteria, the subjects (patients and controls) were ≥ 3 years old and not on antibiotics. The GSD patients also were required to: a) have a genetic diagnosis of GSD and b) be on treatment with UCCS. The healthy controls were recruited by invitation as they came to routine appointments at Santa Cecília Basic Health Unit, Porto Alegre, Brazil. All subjects received a kit and printed instructions for stool collection, storage, and transport. They were also provided with printed instructions to record three days of dietary information. Each participant collected their own frozen fecal sample and three-day dietary record and submitted them to an outpatient clinic during their next routine check-up. Upon returning to the clinic, each participant answered a brief questionnaire about personal features including weight and height, eating habits, intestinal habits, medicines of recent and/or continuous usage and lifestyle. The study protocol was approved by the Ethics Committee of Hospital de Clínicas de Porto Alegre (HCPA). All participants and/or legal guardians signed an informed consent.

As a routine, GSD patients seen at the MGS-HCPA who are on UCCS therapy also receive a multivitamin prescription. Despite optimum dietary treatment other drugs could also be prescribed, mainly for type I patients, such as allopurinol, to prevent gout and urate nephropathy; angiotensin converting enzyme inhibitors, to slow-down or prevent further deterioration of renal function; citrate, to preventing or ameliorating urolithiasis and nephrocalcinosis, in addition to correcting lactacidaemia; statins to treat hypercholesterolaemia [15]; and mainly for Ib patients, G-CSF to treat neutropenia, neutrophil dysfunction and IBD; and the intestinal anti-inflammatory mesalazine (5-amino-salicylic acid), also to treat IBD [16].

### Nutritional assessment, clinical data and statistical analysis

Macro and micronutrients intake by the subjects were estimated from the three-day food records through the Nutribase software (NB16Cloud, CyberSoft, Inc., Phoenix, AZ, USA). The daily nutrient intake of each participant was the sum of the nutrients of each food item. The average of the three-day intake was used for further analysis. Multivitamin consumption and other medications were not included in the nutritional assessment but were considered as variables that potentially were modifying the gut microbial composition, so they were tested by Permutational Multivariate Analysis of Variance. Clinical data, such as IBD and other relevant conditions, were accessed from medical records. BMI-for-age and Z-scores were calculated within the World Health Organization (WHO) AnthroPlus software suite. A qualitative classification for this data followed the WHO criteria [17].

Statistical analysis among the groups was performed using PASW Statistics for Windows software (Vs18.0, 2009, SPSS Inc., Chicago, USA). Numerical variables were compared using the Mann-Whitney U test. Categorical variables were compared using X^2^, Fisher’s exact test or Continuity Correction, when necessary (with statistical significant determined by the threshold p ≤ 0.05). Statistical analyses with the microbiome feature are described below.

### Bacterial DNA extraction, *16S rRNA* gene amplifications and sequencing

The bacterial DNA was isolated from 0.3 mg of frozen faecal sample with QIAamp DNA Stool Mini Kit (Qiagen, Valencia, CA, USA) (Qiagen) according to manufacturer instructions and stored at -20°C until use. The NanoVue system (GE Healthcare, Chicago, IL, USAGE Healthcare) was used to assess the quality of extractions for downstream applications. For the sequencing step, the library was prepared following the procedures described by Barboza et al. [18]. Briefly, region V4 of 16S rRNA gene was amplified with the barcoded bacterial/archaeal primers 515F and 806R [19] in a reaction containing 2U of Platinum Taq DNA High Fidelity Polymerase (Invitrogen, Carlsbad, CA, USA), 4 μL 10X High Fidelity PCR Buffer, 2 mM MgSO4, 0.2 mM dNTPs, 0.1 μM of both the 806R barcoded primer and the 515F primer, 25μg of Ultrapure BSA (Invitrogen, Carlsbad, CA, USA) and approximately 50 ng of DNA template in a final volume of 25 μL. After an initial denaturation step of 5 min at 95°C, 30 cycles of 94°C for 45 s, 56°C for 45 s and 72°C for 1 minute were performed, followed by a final extension step of 10 min at 72°C. After visualization on agarose gel 1.5%, the PCR products were purified with the Agencourt AMPure XP Reagent (Beckman Coulter, Brea, CA, USA) and the final concentration of the PCR product was quantified with the Qubit Fluorometer kit (Invitrogen, Carlsbad, CA, USA) following the manufacturer's recommendations. Finally, the reactions were combined in equimolar concentrations to create a mixture composed of 16S gene amplified fragments of each sample. This composite sample was used for library preparation with the Ion OneTouch 2 System using the Ion PGM Template OT2 400 Kit (Thermo Fisher Scientific, Waltham, MA, USA). Sequencing was performed with Ion PGM Sequencing 400 on the Ion PGM System using Ion 318 Chip v2.

### 16S profiling data analysis

The Fastq files exported from the Ion PGM System were analysed with the BMP Operating System (BMPOS) [20] according to the recommendations of the Brazilian Microbiome Project [21]. Briefly, an Operational Taxonomic Unit (OTU) table was built using reads truncated at 200 bp and quality filtered with a maximum expected error of 0.5. After removing singletons, the sequences were clustered into OTUs at cutoff of 97% similarity, and chimeras were checked and removed to obtain representative sequences for each microbial phylotype. Taxonomic classification was carried out in QIIME version 1.9.1 [22] based on the UCLUST method against the SILVA ribosomal RNA gene database version v132 [23] with a confidence threshold of 80%. Downstream analyses were carried out with dataset rarefied to the minimum library size [24,25] in the R environment [26] using the phyloseq package [27] and vegan package [28]. The online software Microbiome Analyst [29] was used to further detect microbial biomarkers associated with GSD patients. After Cumulative Sum Scaling (CSS) normalization [30], the dataset was analysed by the non-parametric factorial Kruskal-Wallis (KW) sum-rank test followed by Linear Discriminant Analysis [31]. To make sure the biomarkers observed were not only driven by IBD-like patients, we performed one analysis using the full dataset and another analysis excluding all four IBD-like patients and matched controls.

### Faecal calprotectin assay and pH measurement

Frozen faecal samples of patients and controls were thawed and aliquoted at room temperature (20°C) to perform the pH measures and calprotectin assay. To determine the faecal pH, the samples were diluted 1:10 (w/v) in distilled water. After homogenization and incubation for 5 min at room temperature, the faecal pH was measured by an electronic pH-meter (K39-1014B, KASVI, PR, Brazil) three minutes after complete electrode immersion.

The faecal calprotectin was quantified from 100 mg of faecal sample with the RIDASCREEN Calprotectin test (R-Biopharm AG) according to the manufacturer’s instructions. Calprotectin is a calcium-/zinc-binding protein, highly stable and resistant to degradation by intestinal contents (pancreatic secretions, proteases, and bacterial degradation). It is mainly produced by neutrophils in inflammation and has been amply confirmed in intestinal inflammatory diseases [32]. Calprotectin was evaluated to verify gut inflammation across groups and its influence over the number of OTUs. Due to the small sample size of GSD III and IXα, just the subtypes Ia and Ib (groups containing >15% of total sample) were compared. Results for GSD Ia and GSD Ib patients were presented as median (Q1-Q3) and as min-max to GSD III and IXα. To test the correlation among calprotectin and OUT richness, patients who were on mesalazine were excluded from analysis.

## Results

The characteristics of the patients and controls are summarized in Table 1. The nutrient intake varied significantly between groups (S1 Table); the largest variation observed was the higher total carbohydrate and calorie intakes in the GSD group due to UCCS usage. The amount of protein consumed (g) and the number of calories derived from proteins did not differ between patients and controls. However, the percentage of total caloric intake from proteins was lower in patients. Patients ingested less fat (g and Kcal/day) and had a lower percentage of fat in the diet. Regarding micronutrients, patients’ diet was poor in calcium and sodium, and in vitamins B3, H, D and E in comparison to the control group’s diet.

**Table 1:** Sample characterization, analysis of potential confounding variables and their effect on microbial communities.

Variable^1^	Patients (n = 24)	Controls (n = 16)	p-value^1^	Microbial community difference between patients and controls			
				Euclidian Metric		Bray-Curtis Metric	
				r^2^	p-value	r^2^	p-value
Sex (M/F)	14/10	07/09	0.561	0.02942	0.287	0.02964	0.267
Age (yr)	12 (10–19.75)	12.5 (10–23.25)	0.579	0.02895	0.302	0.02775	0.340
Faecal pH	6.23 (5.42–7.16)	7.41 (7.10–7.98)	**0.001**	0.05938	**0.005**	0.08507	**0.001**
Inflammatory Bowel Disease (yes/no)	04/20	00/16	0.136	0.06746	0.009	0.05152	**0.003**
Abdominal pain complaint (yes/no)	09/15	01/15	**0.032**	0.05590	**0.010**	0.04845	**0.009**
Nutritional status* (Obese or Overweight/Normal)	18/06	06/09^†^	**0.044**	0.05199	**0.004**	0.03423	0.121
UCCS intake (g/day)	309.50 (373.7–245.3)	00	**0.001**	0.03698	0.114	0.05594	**0.001**
Drugs (yes/no):							
-Allopurinol	4/20	0/16	0.136	0.02477	0.436	0.02426	0.517
-Antibiotic usage (last 6 months)	10/14	3/13	0.241	0.03047	0.252	0.03200	0.179
-ACE inhibitor	11/13	0/16	**0.001**	0.03351	0.203	0.03919	0.054
-Filgrastim (G-CSF)	5/19	0/16	0.071	0.06654	**0.002**	0.05377	**0.008**
-Mesalazine	3/21	0/16	0.262	0.03089	0.290	0.03389	0.109
-Multivitamin	22/2	1/15	**0.001**	0.04034	0.070	0.05545	**0.003**
-Potassium Citrate	3/21	0/16	0.262	0.02248	0.516	0.02407	0.551
-Proton Pump Inhibitors	2/22	0/16	0.508	0.03068	0.318	0.03087	0.173
-Statins	1/23	0/16	1.000	0.03312	0.286	0.02542	0.486

UCCS: uncooked cornstarch; ACE: Angiotensin-converting-enzyme inhibitor (enalapril maleate); G-CSF: G-colony stimulating factor. Significant (p<0.05) events are highlighted in bold.

^1^ Numeric variables were reported as medians (Q1-Q3). Due to the not-normal distribution, numeric variables were subjected to the Mann-Whitney test. Qualitative variables were reported as absolute frequency and tested by *X*^2^, Fisher’s test or Continuity Correction, as appropriate.

^†^ Data for one control was missing. Weight and height were measured when subjects delivered the sample. In this case, a relative drove the sample to the hospital, thus we were unable to do so.

The intakes of macro and micronutrients were similar among all the GSD types, with some kcal variation from carbohydrate intake due the difference in UCCS consumption among groups (S2 Table).

### Overall 16S rRNA sequencing results, sequence quality control and control for confounding variables

After quality filtering of the 16S rRNA reads, a total of 1,786,582 high-quality sequences longer than 200 bp were obtained. To analyse whether the number of sequences from each sample was representative of the underlying bacterial community, sequence coverage was calculated (S3 Table). An average of 44,664 sequences per sample was obtained with average sequence coverage of 0.99 at the 3% dissimilarity level. This sequencing depth was sufficient to obtain excellent representation of the microbial community in these samples.

Results for suspected confounding variables that potentially were modifying the gut microbial composition are presented at Table 1 and S1 Table. The gut microbial communities were not affected by sex, age, nor the nutritional status of the subjects tested. Faecal pH was lower in patients (6.23) than in controls (7.41), and this variable affected the presence/absence and abundance of the gut microbes, with a reduced OTU count in lower pH. Only 18% of controls (n = 3) and 41% of patients (n = 10) used antibiotics within the 6 months prior to data collection. The use of antibiotics within the 6 months prior to sampling did not affect the presence/absence of microbes (*p* = 0.252) nor microbial relative abundance (*p* = 0.179) in these samples.

### Hepatic GSD is associated with an abnormal gut microbial community

**Fig 1:**
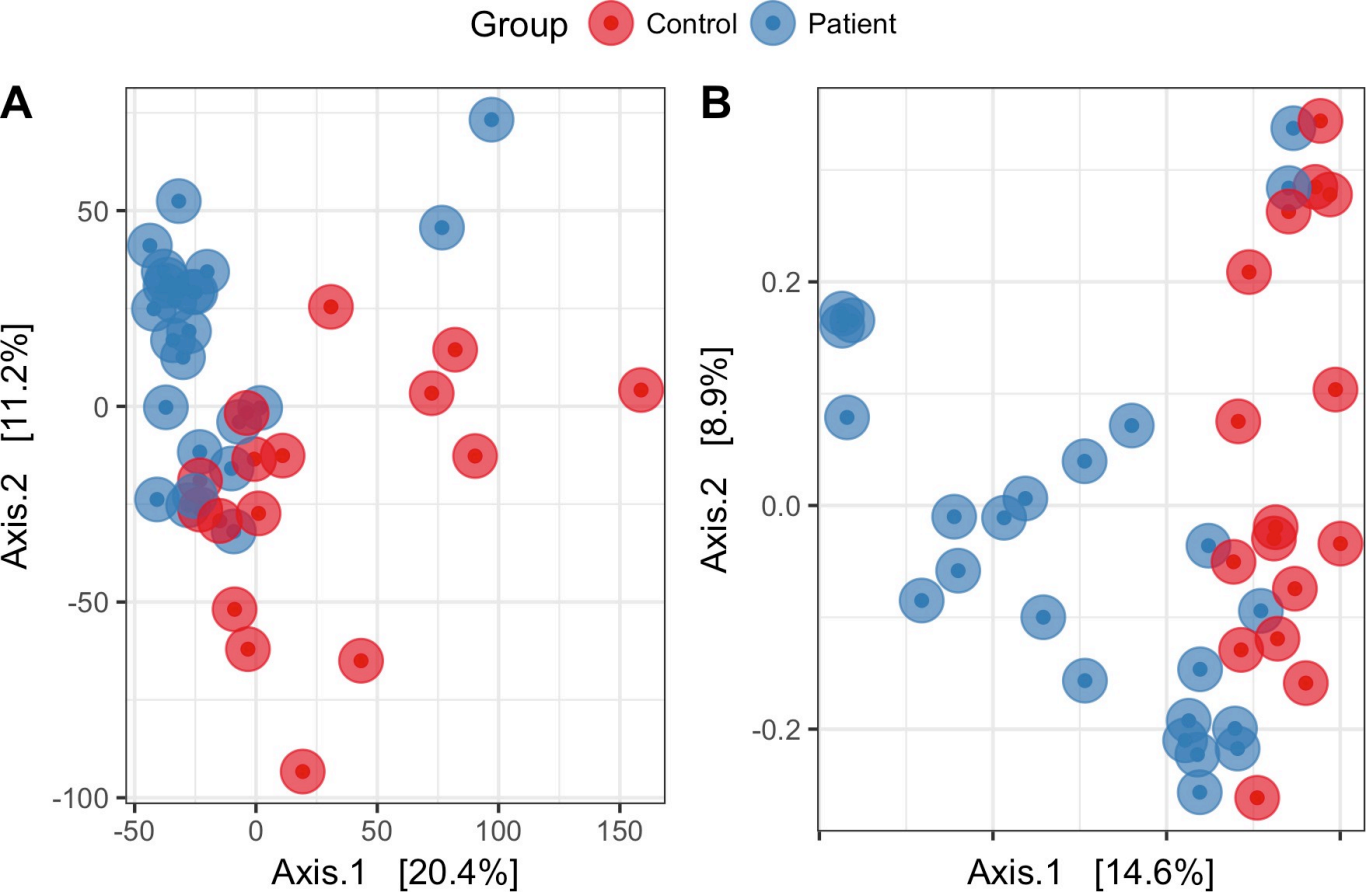
**Principal coordinates analysis (PCoA) based on Bray Curtis distance matrix (A) and Euclidean distance matrix (B) show the separation of gut microbiomes between GSD patients and controls.** Each point represents a microbial community from one subject; colours indicate the treatment.

The analysis of overall microbial community structure revealed significant differences between patients and controls (Fig 1). According to the PERMANOVA, the microbial community structure between patients and controls differed by the presence and absence of taxa (r^2^ = 0.182; p = 0.003) and by their relative abundances (r^2^ = 0.166; p = 0.001). The analysis indicated that the relative abundance of taxa contributed 16% of the variation in the microbial community between patients and controls while the presence/absence of specific taxa contributed 18% to that variation.

**Fig 2:**
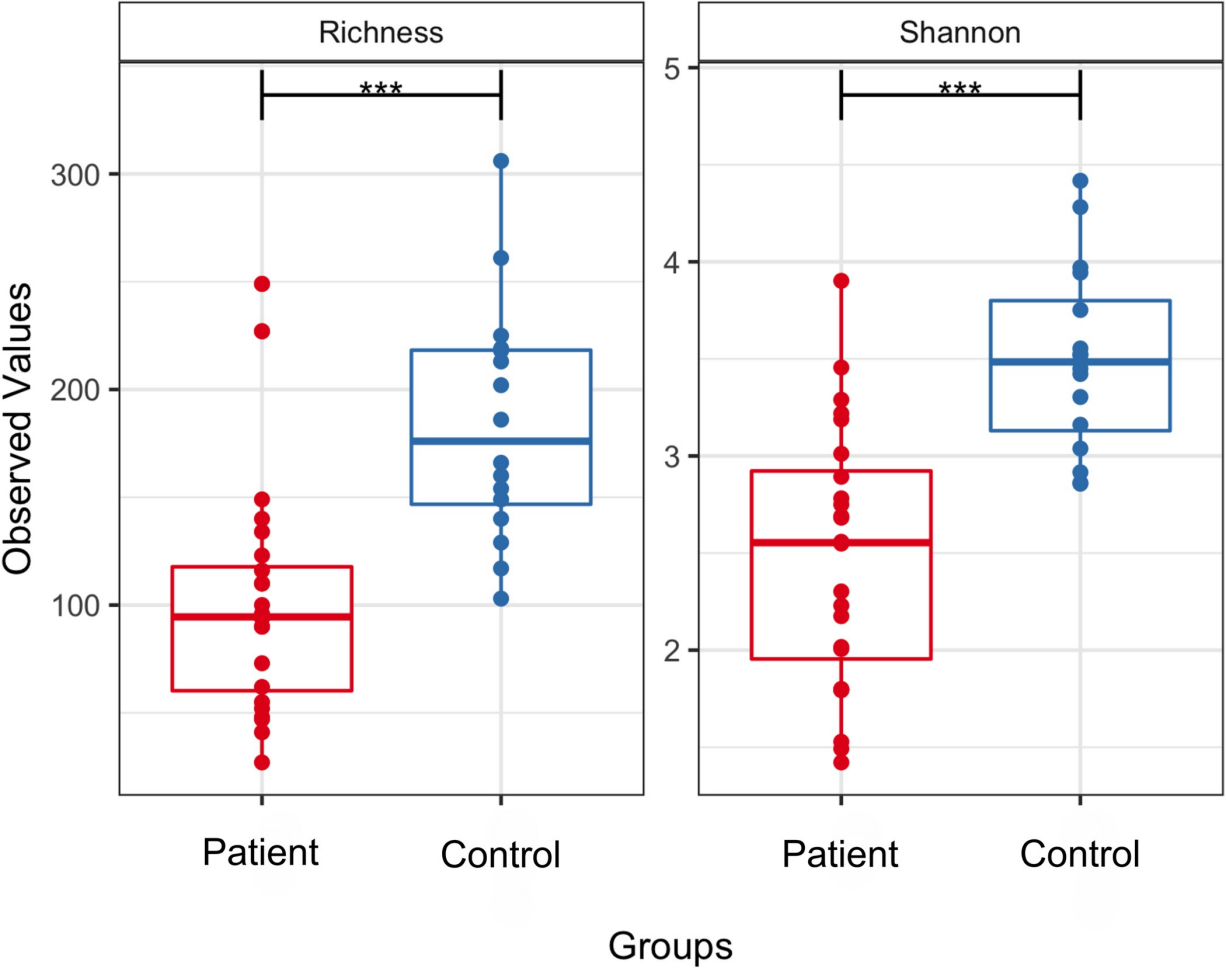
Alpha diversity measurements of microbial communities in the GSD patients and control groups. Each panel represents one alpha diversity measure: Richness = total number of OTUs observed, Shannon = microbial index of diversity. Boxes span the first to third quartiles; the horizontal line within the boxes represents the median. Whiskers extending vertically from the boxes indicate variability outside the upper and lower quartiles. *** indicates a statistical difference between treatments at cutoff *p* ≤ 0.001.

Microbial diversity as measured by richness of OTUs and by the Shannon diversity index also differed significantly (p < 0.01) between patients and controls (Fig 2). On average, control stool samples possessed 184 OTUs while the patients had only 100 OTUs. The average Shannon diversity index was 3.49 and 2.48 in controls and patients, respectively. Together, these beta and alpha diversity analyses indicated that the GSD gut microbiome is characterized by low diversity and distinct microbial structures.

### Defining the main taxa associated with the gut microbiota of patients and controls

**Fig 3:**
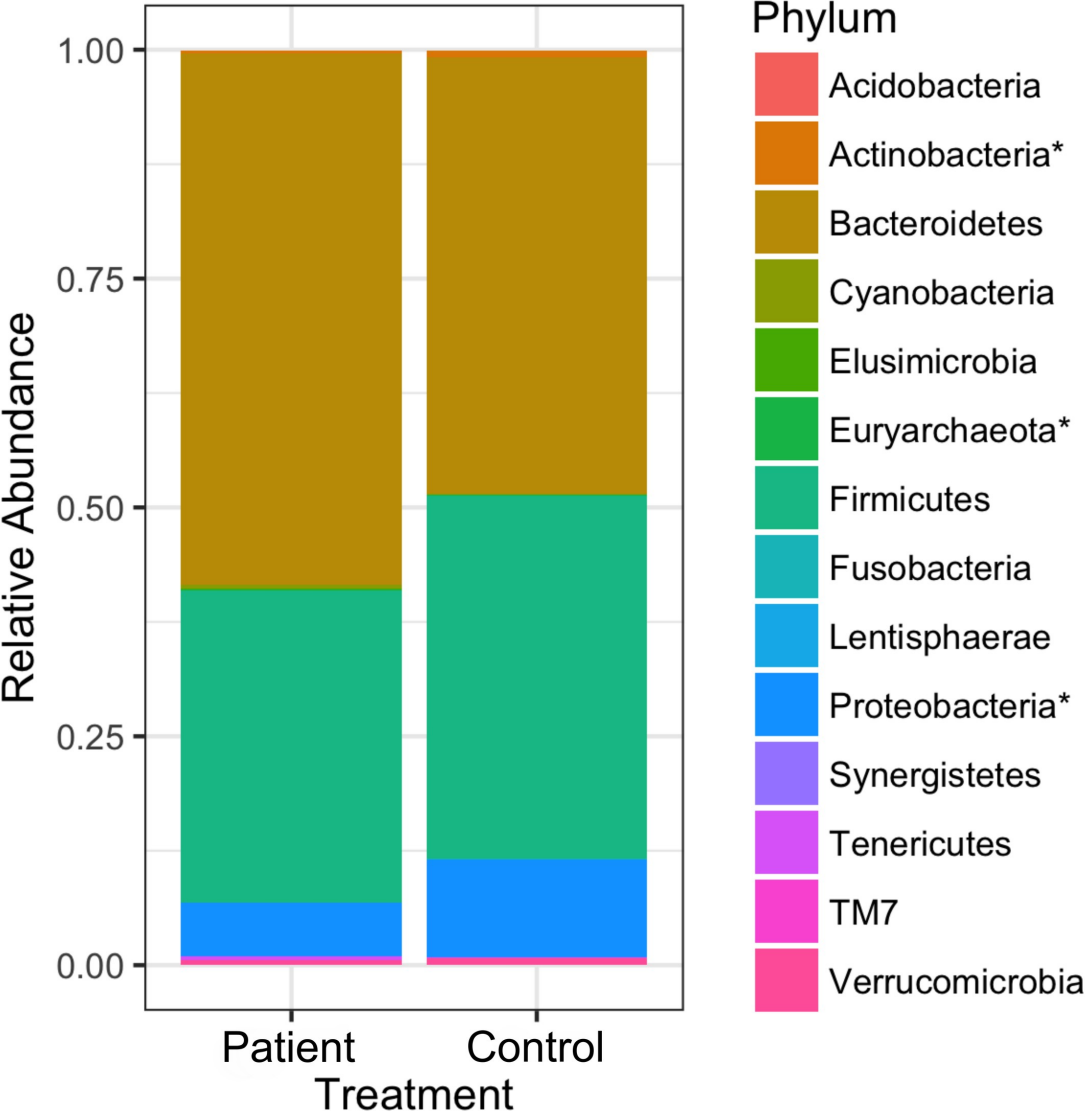
The average relative abundance of phyla found in GSD patients and healthy controls. Phyla followed by an asterisk (*) are different, both in terms of statistics and biological consistency, between patients and controls at *p* and FDR ≤ 0.05: *Euryarchaeota* (LDA score = 1.75), *Actinobacteria* (LDA score = 3.06) and *Proteobacteria* (LDA score = 3.94). *Firmicutes* was marginally significantly different with *p* = 0.064, LDA score = 4.52 and FDR = 0.112.

Specific microbial phylotypes present within the gut community might drive the main differences observed in GSD patients. To find those microbes, biomarker screening analysis was performed at different taxonomic levels. A total of 14 phyla were detected within these samples. However, more than half of the community was dominated by only three phyla: *Bateroidetes* (58% in controls; 47% in patients), *Firmicutes* (34% in controls; 39% in patients) and *Proteobacteria* (5.8% in controls; 10% in patients) (Fig 3). All of the other phyla had very low relative abundances. LEfSe analysis identified three microbial phyla as biomarkers with *Actinobacteria* and *Proteobacteria* overrepresented in patients while *Euryarchaeota* was underrepresented. In particular, *Proteobacteria* presented a very high LDA score (more than 3.9 orders of magnitude), reflecting a marked increase in relative abundance in patients and consistently low abundance in controls. *Firmitutes* had a marginally-significant difference between patients and controls (*p* = 0.043 and LDA score = 4.53 but FDR = 0.07).

At the genus level, nineteen microbial biomarkers were different, both in terms of statistics and biological consistency, between patients and controls (Table 2). Those genera were higher in controls. In patients, those genera were in low abundance and in some cases totally absent. The lack of those microbes might be reflected in the alpha and beta diversity results as mentioned previously (Figs 1 and 2). Besides, *Lactobacillus* and *Escherichia/Shigella* were found to be dominant in patients with a very high LDA score (4.36 and 3.89, respectively), highlighting the biological importance of those microbes in GSD. To remove any biases caused by patients with IBD-like symptoms (n = 4), all IBD-like patients and their respective controls were removed from the dataset and a new biomarker analysis was performed (Table 2). Similar trends as observed within the full dataset were still present in this reduced dataset. However, the *Lactobacillus* genus, found previously in higher abundance in patients was not observed within the dataset without IBD-like patients. On the other hand, *Escherichia/Shigella* was still found to be more abundant in patients than in controls (LDA score = 3.85).

**Table 2:** Microbial biomarkers differentiating patients with hepatic glycogenosis diseases and healthy controls.

Microbial genus	Patients	Controls	*p*-values	FDR	LDA score
	Relative abundance (%)				(log 10)
**Full dataset**	n = 24	n = 16			
*Lactobacillus*	11.31	0.04	0.009	0.025	4.36
*Escherichia/Shigella*	6.70	0.96	0.003	0.013	3.89
*Alistipes*	2.77	9.12	0.005	0.018	-3.22
*Subdoligranulum*	1.59	1.00	0.012	0.029	2.42
*Lachnospiraceae NK4A136 group*	1.44	0.89	0.003	0.013	2.48
*Faecalibacterium*	1.00	3.52	0.016	0.036	-2.98
*Ruminococcaceae UCG 002*	0.98	3.09	0.001	0.007	-2.79
*Bifidobacterium*	0.78	0.19	0.004	0.018	3.1
*Ruminococcus gnavus group*	0.70	0.14	0.007	0.022	3.03
*Phascolarctobacterium*	0.53	1.31	0.015	0.035	-2.56
*Blautia*	0.26	0.53	0.002	0.012	-1.55
*Odoribacter*	0.25	0.53	0.011	0.028	-1.87
*Barnesiella*	0.22	0.98	0.009	0.025	-2.46
*Roseburia*	0.18	1.19	0.002	0.011	-2.78
*Christensenellaceae R 7 group*	0.14	0.80	0.000	0.002	-2.22
*Ruminococcaceae UCG 003*	0.10	0.60	0.000	0.003	-2.27
*Lachnospiraceae UCG 008*	0.04	0.26	0.004	0.018	-1.78
*Ruminococcaceae UCG 005*	0.03	0.25	0.000	0.002	-1.9
*Eubacterium hallii group*	0.02	0.08	0.000	0.002	-1.39
*Anaerostipes*	0.01	0.11	0.001	0.009	-1.55
*Coprococcus 1*	0.01	0.03	0.000	0.005	-0.95
*Family XIII AD3011 group*	0.01	0.05	0.000	0.002	-1.21
*Family XIII UCG 001*	0.00	0.03	0.001	0.007	-1.13
*Methanobrevibacter*	0.00	0.17	0.001	0.007	-1.78
*Ruminococcaceae NK4A214 group*	0.00	0.08	0.001	0.007	-1.5
Dataset without IBD-like patients*	n = 20	n = 14			
*Escherichia/Shigella*	6.47	0.92	0.003	0.027	3.85
*Alistipes*	2.97	9.76	0.008	0.039	-3.28
*Ruminococcaceae UCG 002*	1.12	3.07	0.004	0.028	-1.38
*Bifidobacterium*	0.81	0.08	0.003	0.027	3.2
*Phascolarctobacterium*	0.22	1.38	0.004	0.028	-2.74
*Christensenellaceae R 7 group*	0.17	0.76	0.001	0.016	-2.16
*Blautia*	0.14	0.39	0.001	0.017	-2.08
*Ruminococcaceae UCG 003*	0.11	0.61	0.001	0.016	-2.3
*Roseburia*	0.10	1.15	0.004	0.028	-2.83
*Lachnospiraceae UCG 008*	0.04	0.19	0.011	0.047	-1.57
*Ruminococcaceae UCG 005*	0.03	0.20	0.001	0.016	-1.76
*Eubacterium hallii group*	0.02	0.07	0.000	0.016	-1.32
*Anaerostipes*	0.01	0.07	0.010	0.047	-1.28
*Coprococcus 1*	0.01	0.02	0.008	0.039	-0.77
*Family XIII AD3011 group*	0.01	0.04	0.001	0.017	-1.14
*Family XIII UCG 001*	0.00	0.03	0.001	0.016	-1.15
*Methanobrevibacter*	0.00	0.17	0.003	0.027	-1.81
*Ruminococcaceae NK4A214 group*	0.00	0.08	0.003	0.027	-1.53

* Four IBD-like (Inflammatory Bowel Disease) patients and matched controls were excluded from the dataset to make sure the biomarkers observed were not only driven by these patients.

### Correlations between the gut microbiota, diet, faecal pH and gut inflammation

**Fig 4:**
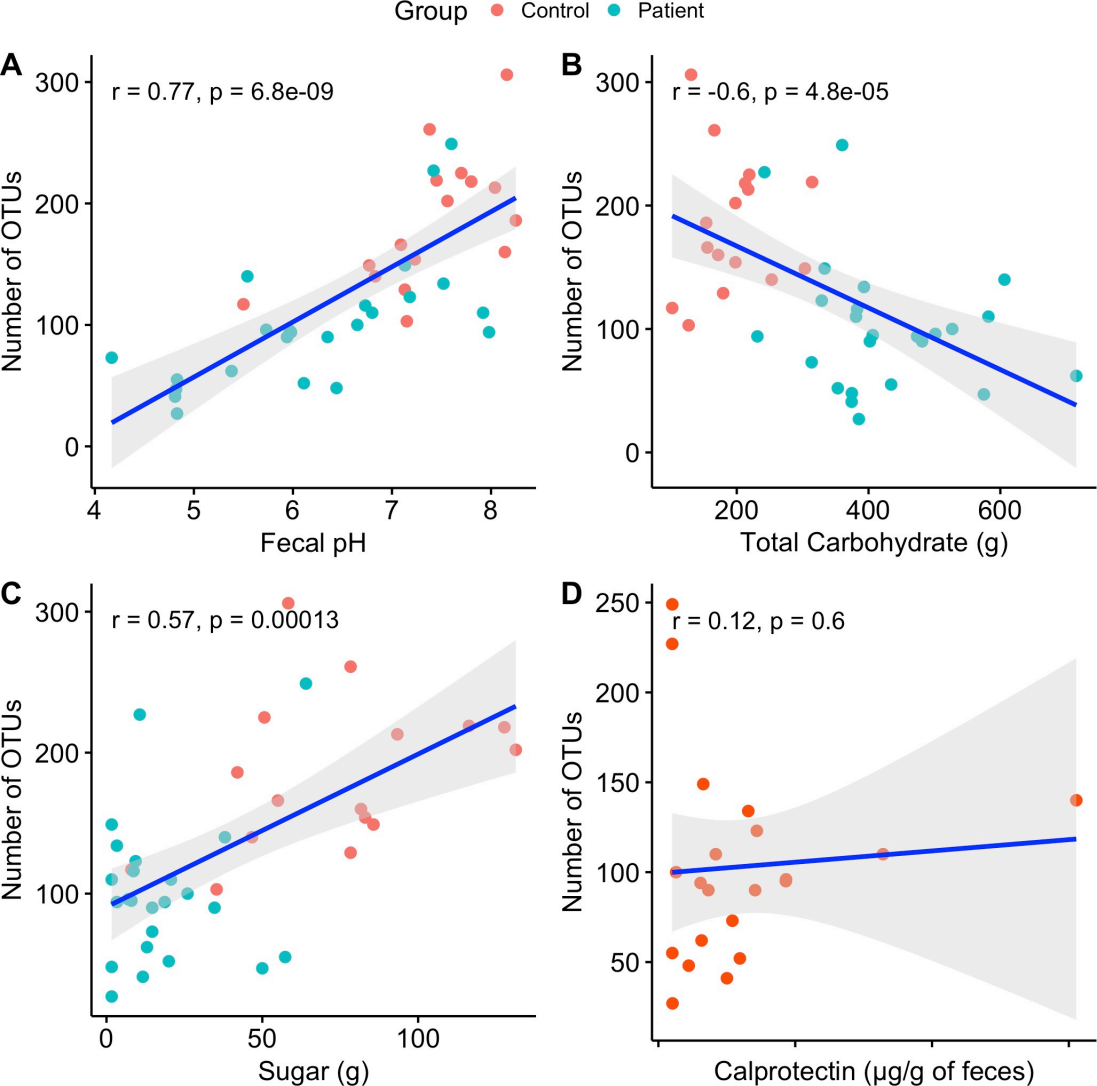
Correlations between the microbiota and diet, faecal pH, and gut inflammation.

Spearman correlations were calculated between the microbiome, diet, faecal pH and calprotectin (Fig 4).

The faecal pH values varied between patients and controls (Table 1), and this was important for shaping their respective differences in gut microbiomes. Differences were determined with the Euclidian distance matrix (for presence/absence of taxa) and the Bray Curtis distance matrix (for relative microbial abundance). Faecal pH was correlated with the total number of microbial OTUs such that higher faecal pH seemed to support more OTUs.

Microbial richness correlated negatively with total carbohydrate but positively with simple carbohydrates (sugar). Calprotectin seemed to have no influence over the microbiome in terms of the number of OTUs (Fig 4). In addition, there was no correlation between this inflammatory marker and gut microbial richness.

## Discussion

This is the first study about the fecal microbiota of GSD patients. In hepatic GSD, high and periodic amounts of UCCS plus dietetic restriction of fast-digestion carbohydrates are the main way to treat the genetic impairment in the glycogenolytic pathway. Our data suggest that the overload of UCCS can lead to low fecal pH by favouring some bacterial genera capable of utilizing complex carbohydrates in detriment of others. The low fecal pH, in turn, also acts as an environmental selection factor to the bacteria in the lumen. Dysbiosis has been associated with IBD and obesity. IBD includes inflammatory bowel diseases of unknown aetiology and has two main forms: ulcerative colitis and Crohn’s disease (CD). CD is a chronic disease that can affect any region in the digestive tract but is more likely to involve the small and large intestines and the perianal region [33]. Enteropathy is related to type I patients, and despite GSD Ib patients are classically described as prone to IBD-Crohn’s-like due the impaired neutrophil activity, this does not explain why patients with GSD Ia also displayed serologic markers altered for IBD, even if asymptomatic [34]. Its not clear if UCCS is the cause of obesity in GSD patients [35], but the microbiome might be associated with it. Here we discuss why the changes in microbiota could be considered as a factor of influence in the phenotype of these patients and why the UCCS usage, even though not exclusively, is an important factor that contribute to that.

Since the introduction of UCCS treatment for GSD, the focus changed from mortality to morbidity and control of long-term complications [36], such as metabolic syndrome and related symptoms [37,38]. GSD type I patients are prone to obesity, and it is suspected that UCCS contributes to the aforementioned features [35,39]. GSD I patients also are subject of heavier doses of UCCS and more restrict diet in comparison with types III and IX [35]. Regarding antibiotics, although its usage clearly drives changes in the gut microbial community, subjects who were treated with antibiotics within 6 months prior to data collection, but not during the study itself, were not affected by the previous antibiotic usage.

We found that the phyla *Actinobacteria* and *Proteobacteria* were overrepresented in patients while the *Euryarchaeota* was underrepresented. The microbiome of GSD patients present low diversity and was highly dominated by *Escherichia/Shigella*.

One possible driver of the differences in gut microbiomes between patients and controls is UCCS overload, which creates an acidic environment [34,40]. In the human body, acids are generated by regular metabolic activities and through the daily intake of food [41]. Fecal pH was lower in patients than controls and stool acidification might lead to an alteration in the relative abundances of fermenting bacteria, decreasing the conversion of unabsorbable starches to short chain fatty acids (SCFAs) [34].

SCFAs, including butyrate, are compounds made by bacteria in the gut that affect several physiologic functions and serve anti-inflammatory roles [42]. Fecal pH was associated with beta diversity and bacterial families belonging to the Clostridia class, an important producer of butyrate in the gut. Several genera of SCFA-producing bacteria—*Coprococcus*, *Blautia*, *Anaerostipes*, *Odoribacter* and *Faecalibacterium*—were decreased in patients. Those genera were also identified in paediatric patients with Crohn’s Disease [43]. Besides, *Coprococcus* and *Faecalibacterium* were found to have significantly low abundance in patients with nonalcoholic fatty liver disease, independently of body mass index and insulin resistance [43].

The bacterial species residing within the mucous layer of the colon may influence whether host cellular homeostasis is maintained or inflammatory mechanisms are triggered. A mutualistic relationship between the colonic microbiota, their metabolic products and the host immune system is likely involved [44]. The phylum *Proteobacteria* was more abundant in patients than in controls while the phylum *Euryarchaeota* was less abundant. *Proteobacteria* is a gram-negative phylum with an outer membrane mainly composed of lipopolysaccharides (LPS), which are known to sustain systemic levels of low-grade inflammation [45]. Higher levels of *Proteobacteria* can be considered a strong marker of dysbiosis [46]. This phylum is prevalent in patients with liver cirrhosis [47]. Several serological markers for IBD were altered in GSD-Ia patients [34], and GSD Ib patients are prone to IBD CD-like. Despite the fact that calprotectin seemed not to influence the number of OTUs gut inflammation (calprotectin >50μg/g) was verified in several patients. GSD type Ib patients have shown a concentration of calprotectin ≤50μg/g, except for one patient, who had an active IBD diagnosed in the same week. This might be due to a remission state and the use of anti-inflammatory mesalazine by these patients.

In general, dysbiosis can be categorized as a) loss of beneficial organisms, b) excessive growth of potentially harmful organisms and c) loss of overall microbial diversity. These three categories often occur at the same time [48]. Dysbiosis has been implicated in a wide range of diseases, including IBD, liver disease and obesity, that are secondary manifestations in GSD patients [49]. The reason for dysbiosis remains unclear, but the overload of UCCS contributes to those characteristics. The food intake records showed a difference in the intake of calories, mainly due to the administration of UCCS in patients, as well as a difference in microbial signature that is known to be related to obesity. It is not known whether these microbiome changes are a cause or a consequence of the pathophysiologies. However, correcting the dysbiosis can improve health in some patients [50–52]. Dysbiosis can also provide biomarkers for disease detection and management [53].

## Conclusion

In this study, we reported significant alterations in the intestinal environments of GSD patients versus healthy controls. Microbiota can be affected by abiotic and biotic factors, namely pH and inflammation, and the differences in these factors between patients and controls might be linked to both genetic disease and UCCS consumption. Several bacterial taxa were different in GSD patients than in controls, and those groups are consistent with the secondary phenotypic manifestations of GSD. The microbiome patterns of these patients may reinforce immune-metabolic pathways that already are altered by genetic impairment, and may also be a factor in the differential individual response to treatment. Patients may gain health and quality of life from the restoration of gut microbial diversity that has been diminished by high UCCS intake. Future research therefore should investigate ways to manipulate the gut microbiome and clarify the possible effects of doing so.

## Supporting information

S1 Table Differences in nutrient mean daily intake between healthy controls and GSD patients and their effect on microbial communities. *Absolute number means that the estimative of ingestion was constant for all the subjects of the group.^1^Mann-Whitney U test. ^2^Bray-Curtis. Significant (p<0.05) events are highlighted in bold.
(PDF)

S2 Table Summary of the finding of the GSD patients (n = 24). OTU: operational taxonomic unit; UCCS: uncooked cornstarch; ACE: Angiotensin-converting-enzyme inhibitor (enalapril maleate); G-CSF: G-colony stimulating factor. ^1^ Numeric variables were reported as Median (Q1-Q3) for GSD Ia and Ib and as Min- Max for GSD III and IXα. Qualitative variables were reported as absolute numbers. P-value was accessed to differences between the groups Ia and Ib. ^†^Calprotectin and number of OTUs for patients on and without mesalazine were reported as Min-Max.
(PDF)

S3 Table Overall description of the 16S rRNA sequencing results among subjects. (PDF)
