# Correction: Hepatic glycogen storage diseases are associated to microbial dysbiosis

**DOI:** 10.1371/journal.pone.0218254

**Published:** 2019-06-06

**Authors:** Karina Colonetti, Bruna Bento dos Santos, Tatiéle Nalin, Carolina Fischinger Moura de Souza, Eric W. Triplett, Priscila Thiago Dobbler, Ida Vanessa Doederlein Schwartz, Luiz Fernando Wurdig Roesch

Fig 3 is incorrect. The authors have provided a corrected version here.

**Fig 3:**
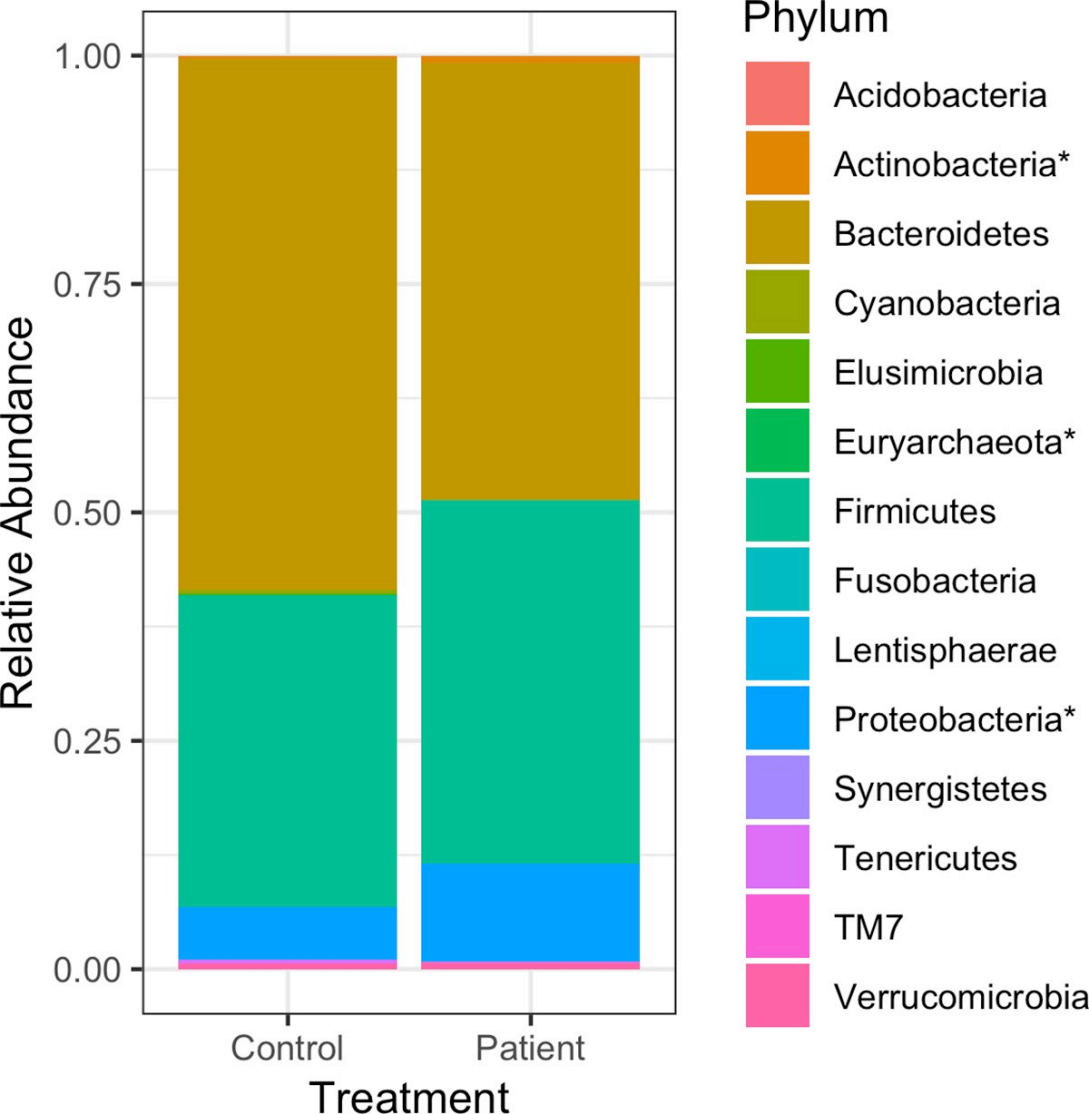
The average relative abundance of phyla found in GSD patients and healthy controls. Phyla followed by an asterisk (*) are different, both in terms of statistics and biological consistency, between patients and controls at *p* and FDR ≤ 0.05: *Euryarchaeota* (LDA score = 1.75), *Actinobacteria* (LDA score = 3.06) and *Proteobacteria* (LDA score = 3.94). Firmicutes was marginally significantly different with *p* = 0.064, LDA score = 4.52 and FDR = 0.112.

Fig 4 is incorrect. The authors have provided a corrected version here.

**Fig 4:**
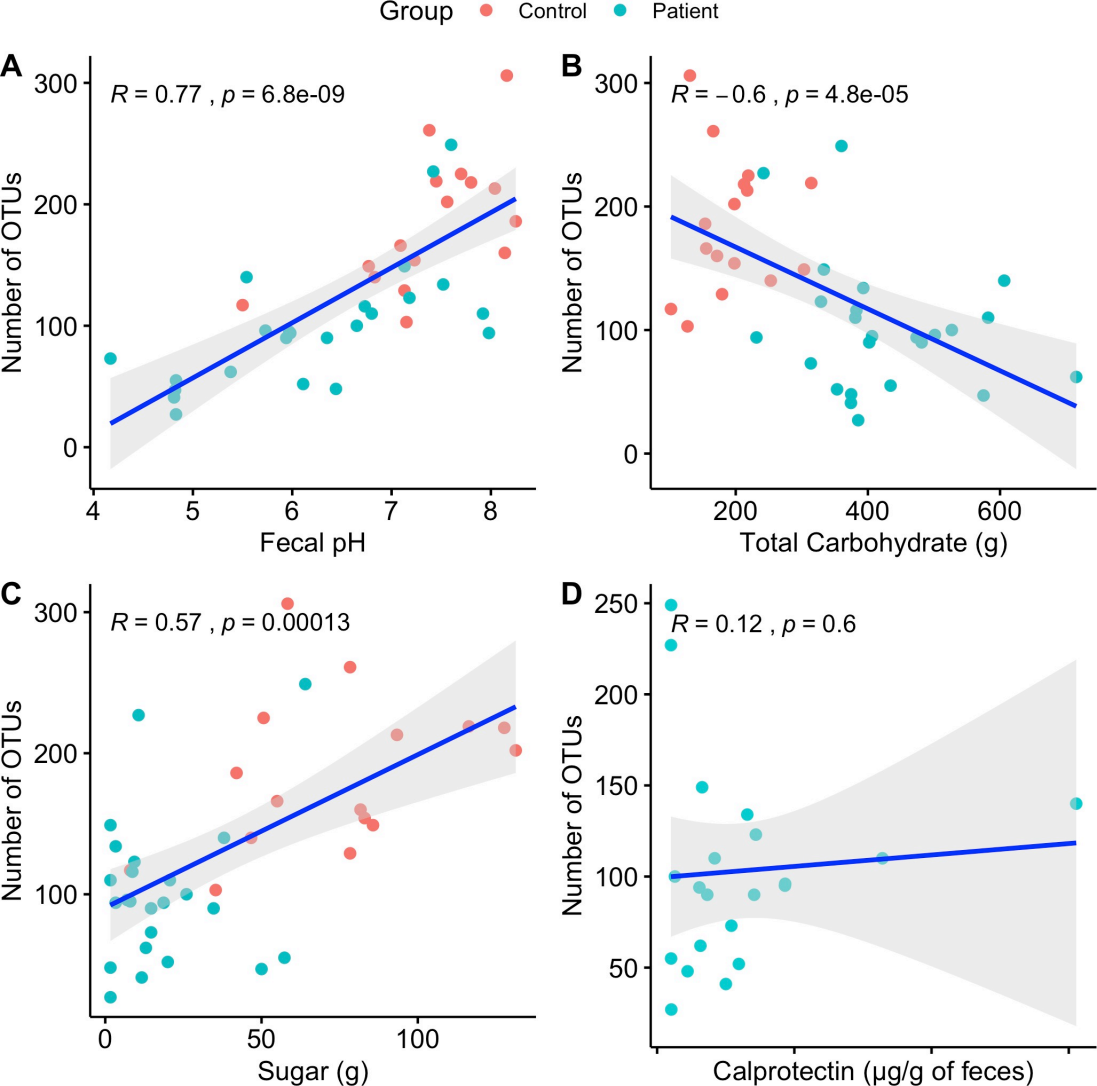
Correlations between the microbiota and diet, faecal pH, and gut inflammation.
