# Supplementary material for: Hepatic glycogen storage diseases are associated to microbial dysbiosis
Source: PLoS One. 2019 Apr 2;14(4):e0214582. doi: 10.1371/journal.pone.0214582 (PMC6445422; doi:10.1371/journal.pone.0214582)
Supplement: S1 Table — *Absolute number means that the estimative of ingestion was constant for all the subjects of the group.1Mann-Whitney U test. 2Bray-Curtis. Significant (p<0.05) events are highlighted in bold. (PDF) [file pone.0214582.s001.pdf]

S1 Table. Differences in nutrient mean daily intake between healthy controls and GSD patients and their effect on microbial communities.

Nutrients	Daily intake Median (Q1-Q3)		p-value[1]	Microbial community difference between treatments (r^2; p-value)[2]
	Patients (n=24)	Control (n=16)		
**Macronutrients**				
Total Calories (kcal total)	2233.83 (1988.16-2867.16)	1520.33 (1143.08-1836.91)	**0.001**	0.045; 0.017
Total Carbohydrate (kcal)	1590.00 (1383.91-2011.00)	747.00 (577.33-827.99)	**0.001**	0.051; 0.006
%Kcal total from carbohydrate	71.66 (67.66-75.25)	49.33 (35.5-55.33)	**0.001**	0.046; 0.018
Total carbohydrate (g)	389.16 (355.00-496.33)	188.66 (154.08-218.58)	**0.001**	0.051; 0.008
* Diet carbohydrate (kcal)	396.55 (240.16-590.53)	747.00 (577.33-827.99)	**0.001**	0.031; 0.159
* Diet carbohydrate (g)	97.33 (58.95-147.50)	188.66(154.08-218.58)	**0.001**	0.033; 0.118
***** Uncooked cornstarch (kcal)	1250.33 (970.19-1494.74)	0.00	**0.001**	0.053; 0.004
* Uncooked cornstarch carbohydrate (g)	309.50 (239.58-368.00)	0.00	**0.001**	0.054; 0.002
Total simple sugars (g)	12.33 (4.25-24.66)	78.33 (47.66-91.41)	**0.001**	0.040; 0.051
Fiber (g)	12.33 (10.66-16.91)	13.00 (5.75-20.25)	0.868	0.025; 0.480
Total fat (kcal)	377.66 (260.41-459.41)	544.83 (367.83-581.91)	**0.008**	0.030; 0.204
% Kcal total from fat	14.66 (10.91-19.66)	34.66 (28.78-38.83)	**0.001**	0.049; 0.007
Fat (g)	40.00 (27.83-48.91)	58.16 (38.66-62.41)	**0.007**	0.031; 0.190
Saturated fat (g)	10.33 (7.75-15.41)	22.00 (14.33-24.91)	**0.001**	0.039; 0.049
Monounsaturated fat (g)	13.33 (8.41-19.08)	16.16 (12.83-20.83)	0.269	0.032; 0.174
Polyunsaturated fat (g)	6.16 (3.91-10.00)	7.33 (5.08-10.58)	0.415	0.018; 0.884
Protein (kcal)	299.33 (238.33-339.91)	254.16 (218.16-365.00)	0.307	0.023; 0.609
%Kcal total from protein	13.50 (10.75-14.66)	19.00 (16.08-25.16)	**0.001**	0.033; 0.116
Protein (g)	71.33 (57.25-79.91)	61.16 (52.41-88.33)	0.314	0.023; 0.586

**Minerals**				
Calcium	206.00 (133.66-306.50)	650.16 (382.33-722.83)	**0.001**	0.033; 0.117
Iron	11.00 (8.91-14.41)	9.33 (7.33-15.08)	0.369	0.024; 0.498
Magnesium	175.00 (122.58-210.00)	193.16 (114.74-242.00)	0.619	0.024; 0.529
Manganese	1.50 (1.00-2.00)	1.83 (1.08-2.25)	0.426	0.021; 0.721
Phosphorus	773.66 (633.16-1001.83)	847.66 (617.41-1088.66)	0.649	0.024; 0.489
Potassium	1492.50 (1084.66-1878.24)	1809.66 (1191.08-2155.66)	0.320	0.024; 0.473
Selenium	81.50 (76.16-93.33)	61.83 (49.25-108.50)	0.143	0.033; 0.138
Sodium	1168.33 (651.99-1765.41)	1702.33 (1305.99-2160.74)	**0.034**	0.037; 0.084
Zinc	9.33 (8.00-10.66)	8.16 (6.00-10.75)	0.139	0.030; 0.221
Chromium	0.00	0.00	-	-
Copper	1.00 (1.00-1.25)	1.00 (0.66-1.00)	0.068	0.035; 0.043
**Vitamins**				
Vit-A (UI)	945.00 (426.50-2758.58)	1297.33 (874.16-5250.16)	0.060	0.021; 0.757
Vit-B1 (mg)	1.00 (0.66-1.00)	1.00 (0.75-1.33)	0.222	0.026; 0.371
Vit-B2 (mg)	1.00 *	1.00 (0.75-1.58)	0.282	0.034; 0.097
Vit-B3 (mg)	16.33 (12.66-19.83)	11.33 (10.00-16.58)	**0.045**	0.025; 0.452
Vit-B5 (mg)	2.83 (2.33-3.66)	3.16 (2.33-4.00)	0.636	0.022; 0.614
Vit-B6 (mg)	1.00 (1.00-1.66)	1.00 (0.41-1.33)	0.173	0.027; 0.328
Total Folate (mcg)	209.83 (159.33-264.83)	243.33 (189.16-373.33)	0.282	0.027; 0.378
Folate, DFE (mcg DFE)	260.33 (190.08-330.00)	281.00 (227.58-434.41)	0.258	0.026; 0.422
Vit-B12 (mcg)	3.33 (2.41-4.50)	3.50 (2.00-4.66)	0.890	0.027; 0.325
Vit-H (mcg)	0.00 (0.00-1.83)	0.00 *	**0.011**	0.037; 0.037
Vit-C (mg)	44.66 (7.50-58.58)	72.83 (26.58-141.33)	0.055	0.027; 0.290
Vit-D (IU)	23.50 (11.83-44.08)	108.66 (51.25-183.08)	**0.001**	0.045; 0.016
Vit-E (IU)	2.33 (1.33-3.58)	3.83 (2.16-7.16)	**0.022**	0.021; 0.716
Vit-K1 (mcg)	39.00 (21.08-54.75)	23.33 (12.75-35.08)	0.090	0.030; 0.187

Significant ($p<0.05$) events are highlighted in bold.

*Absolute number means that the estimative of ingestion was constant for all the subjects of the group.

[1]Mann-Whitney U test

[2]Bray-Curtis
