# Supplementary material for: Hepatic glycogen storage diseases are associated to microbial dysbiosis
Source: PLoS One. 2019 Apr 2;14(4):e0214582. doi: 10.1371/journal.pone.0214582 (PMC6445422; doi:10.1371/journal.pone.0214582)
Supplement: S2 Table — OTU: operational taxonomic unit; UCCS: uncooked cornstarch; ACE: Angiotensin-converting-enzyme inhibitor (enalapril maleate); G-CSF: G-colony stimulating factor. 1 Numeric variables were reported as Median (Q1-Q3) for GSD Ia and Ib and as Min- Max for GSD III and IXα. Qualitative variables were reported as absolute numbers. P-value was accessed to differences between the groups Ia and Ib. †Calprotectin and number of OTUs for patients on and without mesalazine were reported as Min-Max. (PDF) [file pone.0214582.s002.pdf]

S2 Table. Summary of the finding of the GSD patients (n= 24).

Variables[1]	GSD Ia (n=15)	GSD Ib (n=5)	p-value[1]	GSD III (n=1)	GSD Ixα (n=3)
Sex (M/F)	6/8	3/2	1.00	1/0	3/0[2]
Age (yr)	13 (10-20)	10 (4.0-23.5)	0.405	16	11-29
Faecal pH	5.97(5.38-6.80)	5.98 (4.82-6.58)	0.727	7.98	7.18-7.60
Inflammatory Bowel Disease (yes/no)	0/15	4/1	0.116	0/1	0/3
Abdominal pain complaint (yes/no)	5/10	4/1	0.127	0/1	0/3
Nutritional status (Obese+Overweight/Normal)	12/3	5/0	0.539	1/0	0/3
Calprotectin (µg/g)	108.20(65.58-186.30)	On mezalazine= (45.2-285.5)† No mezalazine= (20.65-44.36)	-	61.58	20.18-143.90
Number of OTUs	166.00 (116-208)	On mezalazine= (51-204)† No mezalazine= (61-85)	0.896	176	290-465
Daily Intake					
-Total Kcal	2315.33 (2103.66-2883.33)	2124.66 (1820.33-2795.00)	0.407	2878.33	1668.66-2400
-Total Kcal/kg	39.30 (34.05-51.44)	58 (33.56-81.01)	0.315	36.43	40.68-63.06
-Total Kcal (from carbohydrate)	1662.33 (1514.33-2129.33)	1565.33 (1219.83-1951.00)	0.239	1900.33	935.66-1368.00
-Kcal from diet	387.92(211.42-626.27)	362.81 (197.39-447.57)	0.694	684.70	380.57-1022.22
-Kcal from UCCS	1300 (1108.44-1766.72)	1153.18 (947.90-1576.52)	0.407	1215.62	345.80-951.43
-Total Carbohydrate (g)	406.33 (374.33-527.00)	382.66 (303.00-480.16)	0.315	473.66	242.00-360.00
-Diet Carbohydrates (g)	94.66 (52.33-155.00)	89.66 (48.91-109.83)	0.694	170.66	94.00-269.00
-UCCS Carbohydrate (g)	321.66 (274.0-431.66)	285.00 (234.00-388.00)	0.359	303.00	91.00-235.00
-Protein (g/day)	71 (55-76.66)	70.33 (55.83-107.33)	0.896	102.33	56.67-101
-Fats (g/day)	30.00 (22.00-47.66)	34 (29.0-48.83)	0.513	54.33	40.67-62.67
Drugs (yes/no)					
-Allopurinol	3/12	1/4	1.000	0/1	0/3
-Antibiotic usage (Last 6 months)	5/10	4/1	0.127	1/0	0/3
-ACE inhibitor	10/5	1/4	0.127	0/1	0/3
-Filgrastim (G-CSF)	0/15	5/0	**0.000**	0/1	0/3

-Mesalazine	0/15	3/2	**0.009**	0/1	0/3
-Multivitamin	15/0	5/0	-	1/0	1/2
-Potassium Citrate	3/12	0/5	0.539	0/1	0/3
-Proton Pump Inhibitors	1/14	1/4	0.447	0/1	0/3
-Statins	1/14	0/5	1.000	0/1	0/3

OTU: operational taxonomic unit; UCCS: uncooked cornstarch; ACE: Angiotensin-converting-enzyme inhibitor (enalapril maleate); G-CSF: G-colony stimulating factor.

[1] Numeric variables were reported as Median (Q1-Q3) for GSD Ia and Ib and as Min- Max for GSD III and IXα. Qualitative variables were reported as absolute numbers. P-value was accessed to differences between the groups Ia and Ib.

[†]Calprotectin and number of OTUs for patients on and without mesalazine were reported as Min-Max.
