# Supplementary material for: Hepatic glycogen storage diseases are associated to microbial dysbiosis
Source: PLoS One. 2019 Apr 2;14(4):e0214582. doi: 10.1371/journal.pone.0214582 (PMC6445422; doi:10.1371/journal.pone.0214582)
Supplement: S3 Table — (PDF) [file pone.0214582.s003.pdf]

S3 Table. Overall description of the 16S rRNA sequencing results among subjects.

Subject ID	Number of sequences	Coverage	Observed OTUs
**Controls**			
C01	46,350	0.999	236
C02	49,504	0.999	231
C03	55,620	0.998	384
C04	44,692	0.999	311
C05	54,335	0.999	246
C06	49,219	0.998	341
C07	41,513	0.998	390
C08	58,964	0.999	274
C09	53,734	0.998	397
C10	40,401	0.998	335
C11	53,303	0.998	419
C12	31,736	0.998	205
C13	27,531	0.996	437
C14	23,019	0.998	229
C15	29,800	0.998	270
C16	17,115	0.994	444
**GSD Patients**			
P01	4,724	0.996	51
P02	50,598	0.999	169
P03	44,874	0.999	116
P04	55,299	0.999	166
P05	47,966	0.999	176
P06	20,813	0.997	165
P07	62,846	0.999	85
P08	47,637	0.998	239
P09	39,881	0.999	105
P10	58,674	0.999	138
P11	57,637	0.999	204
P12	59,176	0.999	208
P13	46,914	0.999	175
P14	49,816	0.998	465
P15	68,350	0.999	152
P16	52,118	0.999	61
P17	61,156	0.999	289
P18	58,165	0.999	394
P19	67,259	0.999	290
P20	29,024	0.999	85
P21	37,878	0.999	81
P22	11,018	0.995	155
P23	42,914	0.998	184
P24	35,009	0.998	266
